# *Rv3634c* from *Mycobacterium tuberculosis* H37Rv encodes an enzyme with UDP-Gal/Glc and UDP-GalNAc 4-epimerase activities

**DOI:** 10.1371/journal.pone.0175193

**Published:** 2017-04-12

**Authors:** Peehu Pardeshi, K. Krishnamurthy Rao, Petety V. Balaji

**Affiliations:** Department of Biosciences and Bioengineering Indian Institute of Technology Bombay Powai, Mumbai, India; University of Padova, Medical School, ITALY

## Abstract

A bioinformatics study revealed that *Mycobacterium tuberculosis* H37Rv (Mtb) contains sequence homologs of *Campylobacter jejuni* protein glycosylation enzymes. The ORF *Rv3634c* from Mtb was identified as a sequence homolog of *C*. *jejuni* UDP-Gal/GalNAc 4-epimerase. This study reports the cloning of *Rv3634c* and its expression as an N-terminal His-tagged protein. The recombinant protein was shown to have UDP-Gal/Glc 4-epimerase activity by GOD-POD assay and by reverse phase HPLC. This enzyme was shown to have UDP-GalNAc 4-epimerase activity also. Residues Ser121, Tyr146 and Lys150 were shown by site-directed mutagenesis to be important for enzyme activity. Mutation of Ser121 and Tyr146 to Ala and Phe, respectively, led to complete loss of activity whereas mutation of Lys150 to Arg led to partial loss of activity. There were no gross changes in the secondary structures of any of these three mutants. These results suggest that Ser121 and Tyr146 are essential for epimerase activity of Rv3634c. UDP-Gal/Glc 4-epimerases from other organisms also have a catalytic triad consisting of Ser, Tyr and Lys. The triad carries out proton transfer from nucleotide sugar to NAD^+^ and back, thus effecting the epimerization of the substrate. Addition of NAD^+^ to Lys150 significantly abrogates the loss of activity, suggesting that, as in other epimerases, NAD^+^ is associated with Rv3634c.

## Introduction

### Annotating the molecular function as an important component of functional genomics

The sequence of the *Mycobacterium tuberculosis* (Mtb) H37Rv genome suggests that it encodes ~4000 proteins (http://tuberculist.epfl.ch/) [1]). The functions of many of these proteins have not been determined experimentally so far. Complete characterization of the function of any protein requires the delineation of the biological process in which it participates, its cellular location and molecular function (e.g., reaction catalyzed) (www.geneontology.org). For several Mtb proteins, their cellular location and the biological process with which they are associated have been determined by transcriptomics and/or proteomics [2][3][4][5][6]. For many of these proteins molecular function has been assigned based on similarity in the amino acid sequence to experimentally characterized proteins. Such an assignment need not always be correct since sequence similarity does not necessarily translate to functional similarity. Hence, sequence similarity-based assignment of molecular function needs experimental validation.

### SDR superfamily

Sugar nucleotide epimerases are a class of enzymes that belong to the extended SDR subfamily of the short chain dehydrogenase reductase (SDR) superfamily [7]. The other classes of enzymes in this superfamily include SDR type dehydratases, isomerases and decarboxylases. Members of this extended SDR subfamily have a Rossmann fold domain in the N-terminal region and this domain contains the NAD(P) binding motif GxxGxxG. The catalytic site consists of the motif YxxxK in most of the SDR enzymes [8][9]. In general, dehydratases and epimerases have similar sequences and it is not possible to unambiguously annotate them by sequence comparison alone. Also, reaction mechanisms of epimerases and dehydratases are similar to the extent that both involve proton abstraction and transfer to NAD as the first step of reaction [10].

A large number of UDP-Gal/GalNAc epimerases have been characterized with respect to their substrate specificity. Notably, they differ from each other in the utilization of N-acetylated derivatives and are accordingly classified into three types viz., Type I, Type II and Type III. Type I enzymes (e.g., *E*. *coli* GalE) utilize only UDP-Glc/Gal as substrates whereas Type III enzymes (e.g., *P*. *aeruginosa* Gne) use only UDP-GlcNAc/GalNAc as substrates. Type II (e.g., *Homo sapiens* Gne) enzymes can utilize both these as substrates [11][8].

### Sequence similarity-based assignment of molecular function of Rv3634c

The molecular function of Rv3634c has not yet been determined experimentally. Rv3634c has 314 amino acids and of these, the sequence of residues 3–241 aligns with those of NAD-dependent epimerase / dehydratase family of proteins (accession no. PF01370) in the Pfam database (pfam.sanger.ac.uk) suggesting that Rv3634c belongs to this family. When the Mtb genome was sequenced and putative ORFs annotated, it was found that the Mtb genome has four sequence homologs of dTDP dehydratase (also called as *RmlB*); *Rv3634c* is one of these four homologs [1][12]. It has been suggested that *Rv3634c* encodes UDP-Gal 4-epimerase and not RmlB, based on its N-terminal sequence similarity to GalE from *M*. *smegmatis* [12][13].

**Fig 1:**
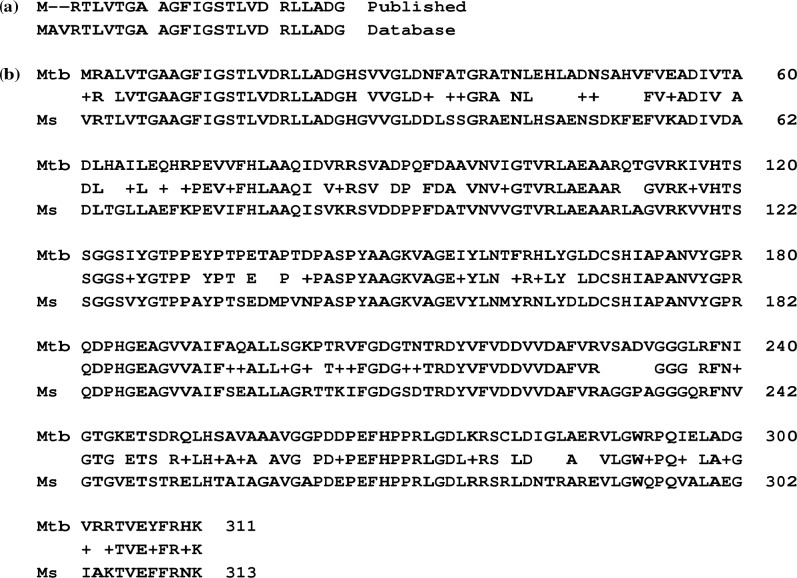
(a) Comparison of the published N-terminal sequence of UDP-glucose 4-epimerase [13] with that of nucleoside diphosphate sugar epimerase in the database (MSMEG_6142). Both are from *M*. *smegmatis*. (b) Alignment of Rv3634c (labelled as Mtb) and MSMEG_6142 (315 residues; labelled as Ms) obtained by pair-wise BLAST option at NCBI with default values for all the parameters. Few residues at the termini are excluded from the alignment. These are (i) C-terminal His-Thr-Asp of Rv3634c and (ii) N-terminal Met-Ala and C-terminal Ser-Gln of MSMEG_6142.

The sequence of the N-terminal 24 amino acid residues of UDP-Glc 4-epimerase of *M*. *smegmatis* has been published (Fig 1A) [13]. The entry in the NCBI (www.ncbi.nlm.nih.gov) protein sequence database (MSMEG_6142) which probably corresponds to the experimentally characterized *M*. *smegmatis* UDP-Glc 4-epimerase [13] is annotated as nucleoside diphosphate sugar epimerase. This entry has no associated publication. Rv3634c and MSMEG_6142 have highly similar primary structures: they share 71% identity and 84% similarity, and there are no gaps in their pair-wise alignment (Fig 1B).

### Objective of the present study

*Mycobacterium tuberculosis* is reported to have glycoconjugates on its cell surface as well as in the cytoplasm [14]. Many enzymes involved in the biosynthesis of glycoconjugates like arabinogalactan, lipoarabinomannan and phenolic glycolipids have been experimentally identified so far [14]. Some of them include rmlC [15], glmU [16] and pimA [17]. The present study was undertaken with the objective of experimentally determining if the protein encoded by *Rv3634c* has epimerase activity. The gene *Rv3634c* was cloned and expressed in *Escherichia coli*. Purified recombinant Rv3634c was indeed found to have epimerase activity when tested with UDP-Gal/Glc and UDP-GalNAc as substrates. Single point mutants S121A and Y146F were found to be inactive whereas K150R is partially active. Secondary structures of these three mutants are similar to that of the wild type as inferred from CD spectroscopy. It is conceivable that *Rv3634c* is part of the glycosylation machinery since Mtb has a variety of cell surface glycoconjugates and some of its proteins are known to be glycosylated.

## Materials and methods

### Materials

DH5α and BL21(DE3)pLysS strains of *E*. *coli* are maintained as laboratory stocks. Plasmid pYUB28b and strain *Mycobacterium smegmatis* mc^2^ 4517 were a kind gift from Dr. Ghader Bashiri, New Zealand [18]. Oligonucleotide primers were synthesized on order, in desalted form, by Sigma–Aldrich Chemicals Pvt. Ltd., India.

Restriction endonucleases, T4 DNA ligase, DNA molecular weight marker, dNTPs, *Pfu* DNA polymerase, polynucleotide kinase, DNA ligase and *Taq* DNA polymerase were procured from MBI Fermentas, Germany. Protein molecular weight markers were procured from Bangalore Genei or Sigma Aldrich Chemicals Pvt. Ltd., India. Agarose, calcium chloride, PMSF, DTT, acrylamide, N,N^′^-methylene-acrylamide, Coomassie brilliant blue-G and -R, APS, TEMED were procured from Sigma-Aldrich Chemicals Pvt. Ltd., India. Thrombin was purchased from Novagen, USA. *Streptococcus thermophilus* UDP-Gal 4-epimerase was purchased from Calbiochem, USA. Antibiotics and IPTG were from MP Biomedicals, India or Himedia, India. Culture media were from Himedia, India. Qiaexpress Ni-NTA spin kits were procured from Qiagen, USA. All other chemicals were of analytical reagent grade procured from Sisco Research Laboratories, India or Himedia, India. Protease inhibitor cocktail tablets were purchased from Sigma Aldrich Chemicals Pvt. Ltd., India.

### Methods

#### DNA amplification of the gene *Rv3634c*

The ORF *Rv3634c* was PCR-amplified from the genomic DNA of *Mycobacterium tuberculosis* H37Rv kindly provided by Dr. Vinay Nandicoori, National Institute of Immunology, New Delhi. The PCR amplification reactions were carried out with *Pfu* DNA polymerase according to manufacturer’s protocol using gene specific primers, having appropriate restriction sites for cloning. Four extra nucleotides were added at the 5′ end of each restriction site to facilitate proper digestion by restriction endonucleases (S1 Table). Amplification was confirmed by agarose gel electrophoresis. Restriction analysis was used to ascertain that the amplicons have expected restriction sites.

#### Cloning of the wild type gene *Rv3634c*

*Rv3634c* was first cloned in pBSK- in HindIII and BamHI sites. For this, ORF *Rv3634c* was amplified from *Mycobacterium tuberculosis* H37Rv genomic DNA using the primers PSP021 and PSP024. HindIII and BamHI digested amplicon was ligated to HindIII and BamHI digested pBSK- plasmid. The recombinant plasmid was named as pBSK3634. Rv3634c was sub-cloned from pBSK3634 into pYUB28b at NdeI and BamHI sites to express the ORF as N-terminally His-tagged protein. This plasmid was named as pYH3634. *Rv3634c* was also cloned in a direction opposite to that of translation. For this, the HindIII-BamHI fragment was excised from pBSK3634 and ligated to pYUB28b at HindIII and BamHI sites. This plasmid was named as pY3634Rev. All plasmids were maintained in *E*. *coli* DH5α and isolated using standard protocol [19].The sequences of the inserts were confirmed by Macrogen plasmid sequencing service, South Korea.

#### Expression of Rv3634c in *E*. *coli*

To express the recombinant proteins in *E*. *coli*, chemically competent cells of strains BL21(DE3)pLysS, BL21(DE3), BL21-CodonPlus(DE3)-RIPL, OverExpress C41(DE3), or OverExpress C43(DE3)pLysS were transformed with recombinant plasmids using standard protocols [19], and plated on LB+hygromycin agar plates. Transformants were picked and cultured overnight at 37^°^C under shaking conditions (200rpm). Cells were grown on LB broth for IPTG induction or on Auto-Induction Medium (AIM-LB broth) with trace elements. Media were inoculated with 1–2% of overnight culture. In case of LB broth cultures, cells were allowed to grow for 2-3h at 37°C and then induced with varying concentrations of IPTG. Induced cultures were further grown at various temperatures for optimal protein expression and solubility. Auto-Induction Medium cultures were allowed to grow and autoinduce at 25^°^C for 12 h. In all cases, cells were harvested and cell pellets were stored at -80^°^C until further use. To check the expression of the proteins, an aliquot of cell pellets was re-suspended in water and heated at 95^°^C for 5min in water bath. The lysed cells were centrifuged at 8,000×*g* for 10min at 4^°^C to separate cell debris from the supernatant.

To separate soluble and membrane fractions, cell pellets were treated with the lysis buffer 50mM Tris-HCl pH 8.0, 500mM NaCl, 0.5% TritonX100 containing one tablet of protease inhibitor cocktail for 30min at room temperature. After this, the lysate was homogenised by sonication on ice at 30% amplitude with 20x5s pulses in 5s interval. Lysates were centrifuged at 20,000×*g* at 4^°^C for 20min. The supernatant was collected in fresh tubes and pellets were resuspended in lysis buffer. Bradford assay was done according to the standard protocol to determine protein concentration in each fraction. Equal amounts of protein samples were loaded on a 15% SDS PAGE gel and run at 80V following standard protocol [19]. Aliquots of supernatant and re-suspended pellet fractions were either used fresh for assaying enzyme activity or stored at -20^°^C until further use.

#### Expression of Rv3634c in *Mycobacterium smegmatis*

For expression of recombinant Rv3634c in *Mycobacterium smegmatis* mc^2^ 4517, electrocompetent cells were transformed with pYH3634, pYUB28b or pY3634Rev by electroporation [20][18]. Briefly, *M*. *smegmatis* culture was grown to saturation (4–5 days) at 37°C by inoculation from a glycerol stock into Middlebrook 7H9 broth containing 0.1% Tween 80 and ADC supplement (7H9-ADC-TW) with 50 μg/ml kanamycin. 1 ml of the saturated culture was then used to inoculate 100 ml of fresh 7H9-ADC-TW medium with 50 μg/ml kanamycin and grown to OD_600_ ~0.7 (15-20h) at 37°C. Cells were harvested and washed thrice with equal volumes of 10% ice-cold glycerol by centrifugation at 18,000×*g* for 10 min. Finally, cell pellets were re-suspended in 1/500^th^ volume of ice-cold glycerol and stored at -80°C until further use. For electroporation, 40 μl of this competent cell suspension was taken and 2 μl of DNA and 260 μl of 10% glycerol were added to it. After mixing by pipetting, the mixture was placed on ice for 1 min and then transferred to 0.2 cm Bio-Rad cuvette. The cuvette was placed in a Bio-Rad Gene Pulser and electroporation was done with these settings: voltage 2500 V, resistance 1000 Ω and capacitance 25 μF for 15–25 msec time constants. Transformed cells were immediately suspended in 1ml of 7H9-ADC-TW broth and incubated for 3 h with constant shaking. The cells were then plated on 7H10-OADC-glycerol agar plates containing 50 μg/ml each of kanamycin and hygromycin.

Transformants were inoculated into 10 ml of 7H9-ADC-TW medium containing 50 μg/ml each of kanamycin and hygromycin and allowed to grow at 37°C at 200 rpm for 48 h. 100 ml of 7H9-ADC-TW medium or ZYM-5052/0.1% Tween 80 auto-induction medium with 50 μg/ml each of kanamycin and hygromycin in 500 ml baffled flasks were inoculated with 1% of the saturated culture and incubated at 37°C at 200 rpm for 12 h. Cultures with 7H9-ADC-TW medium were induced with 1 mM IPTG, leaving 1 flask uninduced to act as negative control. After induction, incubation was continued under same conditions for 72 h. For culture with ZYM-5052/0.1% Tween 80 auto-induction medium, growth was followed up to 8 days. Aliquots were taken after every 6–12 h for induction profile experiments as well as growth curve. For checking the expression and activity, cells were lysed by incubation for 1h at room temperature with lysis buffer containing 50 mM NaH_2_PO_4_ pH 8.0, 500 mM NaCl and 0.5% Triton X-100 followed by sonication on ice at 40% amplitude in 10 s pulses for 5 min with 5 s intervals. Cell debris were separated from soluble fraction by centrifugation at 20,000x*g* for 30 min at 4°C. Epimerase activity in supernatants was measured using GOD-POD assay. Supernatant and pellet fractions were analysed by SDS-PAGE.

#### Protein purification

To purify the protein at preparatory scale, cell pellet obtained from 2.5 L culture was re-suspended into 500 ml of lysis/binding buffer (50 mM Tris-HCl pH 8.0, 500 mM NaCl, 5% glycerol, 0.5% Triton X-100) along with one tablet of protease inhibitor cocktail (Sigma-Aldrich) and lysed by sonication on ice at 40% amplitude with 5 s On and 1 s Off cycles for a total of 10 min. Cell debris was removed by centrifugation at 6500×*g* for 60 min at 4°C. The soluble fraction so obtained was loaded onto 5ml of packed Ni-NTA agarose resin pre-equilibrated with 2 column volumes of binding/lysis buffer at 4°C. Flow rate was maintained at 0.2 ml/min. All steps were carried out at 4°C. Unbound fractions were discarded and the column was washed with 150 ml of wash buffer I (20 mM imidazole, 50 mM Tris-HCl pH 8.0, 500 mM NaCl, 5% glycerol), 150 ml of wash buffer II (50 mM imidazole, 50 mM Tris-HCl pH 8.0, 500 mM NaCl, 5% glycerol) and 15 ml of wash buffer III (80 mM imidazole, 50 mM Tris-HCl pH 8.0, 500 mM NaCl, 5% glycerol). The bound protein was eluted using 20 ml of elution buffer (200 mM imidazole, 50 mM Tris-HCl pH 8.0, 500 mM NaCl, 5% glycerol). The eluted protein was concentrated using 10 kDa MWCO centrifugal filter (Amicon Ultra) and buffer exchanged to FPLC buffer (20 mM Tris, pH 8.0, 100 mM NaCl). 2 ml of the concentrated protein was loaded onto a size exclusion column (Superdex 200 100 cm 16/600 GL GE Healthcare) pre-equilibrated with FPLC buffer. The protein was eluted with FPLC buffer at a flow rate of 1 ml/min and 500 μl fractions were collected. Peak fractions of the expected size were pooled and concentrated using a 10 kDa MWCO centrifugal filter and used freshly or stored at -20°C in the form of small aliquots until further use. All expression and purification profile experiments were done at least three times.

#### GOD-POD assay for UDP-Gal/Glc epimerase activity

Glucose oxidase-horseradish peroxidase (GOD-POD) coupled assay was used to assay the activity of His-tagged Rv3634c (H3634) expressed from pYH3634. This is a convenient spectrophotometric method to determine the amount of glucose released from acid hydrolysis of UDP-Glucose present in the reaction mixture (S1 Fig). The protocol was followed as reported [21] except that 10 mM Tris-HCl, pH 8.0 was used as the reaction buffer. Briefly, 0–2 mM UDP-Gal or UDP-Glc dissolved in 10 mM Tris-HCl, pH 8.0 was incubated with different amounts of cell lysates or purified protein at 37^°^C for varying time intervals in a total reaction volume of 44 μl. Reactions were quenched and acid hydrolysis was done using 7 μl of 0.4 N HCl and heating at 100°C for 6 min. The mixture was neutralized using 7 μl of 0.4 N NaOH. 15 μl aliquots were taken from each reaction mixture and applied to a clean 96 well microtitre plate. To detect the released glucose, 135 μl of reaction cocktail containing 22 U/ml glucose oxidase, 7 U/ml of horseradish peroxidase and 0.3 mg/ml of O-dianisidine in sodium acetate buffer (pH 5.5) were added to each well of the microtitre plate and incubated for 1 h at 37^°^C. Reaction was quenched and colour developed by adding 200 μl of 6 N HCl per well. Absorbance was read at 540 nm. The assay was standardized using glucose and UDP-glucose. Commercially available UDP-Gal 4-epimerase from *Streptococcus thermophilus* (Calbiochem) was used as a positive control for this assay (1.4 mU per reaction). The assay was performed with soluble fractions of induced and uninduced cell lysates as well as purified H3634. Denatured cell lysates were used as negative control. All reactions were performed in duplicates.

#### Enzyme assay by HPLC

To confirm the UDP-Glc/Gal and UDP-GlcNAc/GalNAc epimerization activity of H3634, HPLC was used to detect nucleotide sugars. 100mM potassium phosphate with 8mM tetrabutylammonium hydrogen sulphate, pH 6.4 was used as mobile phase [22]. Zorbax SB-C18 column (4.6 x 250 mm, 5 μm) was equilibrated with the mobile phase by passing a minimum of 300 column volumes of the mobile phase. 20 μl of samples were injected and run at 40°C at a flow rate of 1 ml/min. UV detector was used to detect nucleotide sugars by their absorbance at 254 nm. To standardize the experiment, known concentrations of UDP-Glucose, UDP-Galactose, UDP-GlcNAc, UDP-GalNAc were run on the column. In order to analyse the reactions carried out by H3634, purified H3634 was incubated separately with each of the substrates at 37°C for 2–10 h in a reaction volume of 44 μl. Reactions were quenched by heating the reaction mixture to 100°C for 10 min in a water bath. The precipitated proteins were removed by centrifugation at 24,000×*g* for 10 min and 20 μl of the supernatant was injected into the HPLC column. All the mobile phases were filtered through 0.22 μm filters and degassed using a vacuum pump. All reactions were performed at least twice.

#### Site directed mutagenesis

The *Rv3634c* gene was mutated by whole plasmid PCR based site directed mutagenesis method using specific primers (S1 Table) to generate 3 single point mutants where Ser121 is replaced by Ala, Tyr146 is replaced by Phe or Lys150 is replaced by Arg. Each 50 μl PCR mixture contained 0.2 mM dNTPs, Pfu buffer/KOD Hotstart buffer, 2.5 mM MgSO_4_, 5% DMSO, 5% glycerol, 150 ng primers, 20 ng template DNA (pYH3634), 1U Pfu polymerase / KOD Hotstart polymerase. Denaturation was at 95°C for 30 s or 98°C for 45 s, annealing at 60°-70°C for 30 s and extension at 72°C for 3 min for 30 cycles. Final extension was done at 72°C for 10 min. 20 μl of the PCR product was treated with 1 U polynucleotide kinase in presence of 1X forward buffer A, at 37°C for 2 h and then with 1 U of T4 DNA ligase in presence of 1X ligation buffer at 22°C for 2 h. Finally, ligated products were treated with DpnI for 2 h at 37°C and used to transform ultracompetent cells of *E*. *coli* DH5α. The transformation cell suspension was spread on LB agar plates containing 50 μg/ml of hygromycin. Transformant colonies were picked after 14 h incubation at 37°C and transferred to fresh LB agar + hygromycin plates. Mutant plasmids from transformed cells were sequenced from both ends of the gene *Rv3634c* using T7 promoter and T7 terminator primers. The sequencing service was provided by Macrogen, South Korea. Plasmids containing desired mutations, as confirmed by DNA sequencing, were used to transform competent *E*. *coli* BL21(DE3)pLysS cells. Mutant proteins were expressed and purified as described for the wild type protein (WT).

#### Secondary structure analysis using circular dichroism

3–5 μM of each protein in 100 mM NaCl, 20 mM Tris, pH 8.0, 5% glycerol was taken in a cuvette of 1 mm path length and CD spectra were acquired from 260–198 nm wavelength range. The molar residual ellipticity of each protein was calculated and the spectra of mutant proteins were compared with that of WT in order to determine changes, if any, in the gross secondary structural properties in the mutants as compared to the WT. Deconvolution analysis was done to determine the percentage of each secondary structure type in the protein. Readings from at least two spectra were used to calculate molar ellipitcity.

#### Oligomerization status of the WT and mutant proteins

The oligomerization status of proteins were determined using size exclusion column (Superdex 200, 100c m, 16/600 GL GE Healthcare) chromatography using the FPLC buffer. The column was calibrated using Low Molecular Weight calibration kit which includes conalbumin, ovalbumin, carbonic anhydrase, ribonuclease A and aprotinin. Blue dextran was used to find the void volume. The partition coefficient *K*_*av*_ was calculated using the equation

$$K_{av}=\frac{V_{e}-V_{0}}{V_{c}-V_{0}}$$

Where *V*_*e*_ is the elution volume of the protein, *V*_0_ is the void volume of the column and *V*_*c*_ is the geometric volume of the column. A plot of *K*_*av*_ against the molecular weight of proteins was used to calculate the molecular weight of unknown protein samples.

## Results

### Cloning, expression and purification of recombinant Rv3634c

*Rv3634c* is a 945 bp ORF located on the complementary strand of *Mycobacterium tuberculosis* H37Rv genome and codes for a protein of length 314 residues. In order to express Rv3634c with a His-tag at the N-terminus, *Rv3634c* was cloned in pYUB28b, a shuttle vector for *Mycobacterium smegmatis* and *E*. *coli* to give the plasmid pYH3634 (S2 Fig). The plasmid pY3634Rev containing the gene *Rv3634c* in the reverse orientation was also generated in order to act as a negative control for protein expression. For this, the gene *Rv3634c* was cloned between HindIII and BamHI in pYUB28b such that its reading frame is opposite to the direction of transcription. This resulted in the plasmid pY3634Rev which cannot express Rv3634c even though it contains the gene *Rv3634c*.

**Fig 2:**
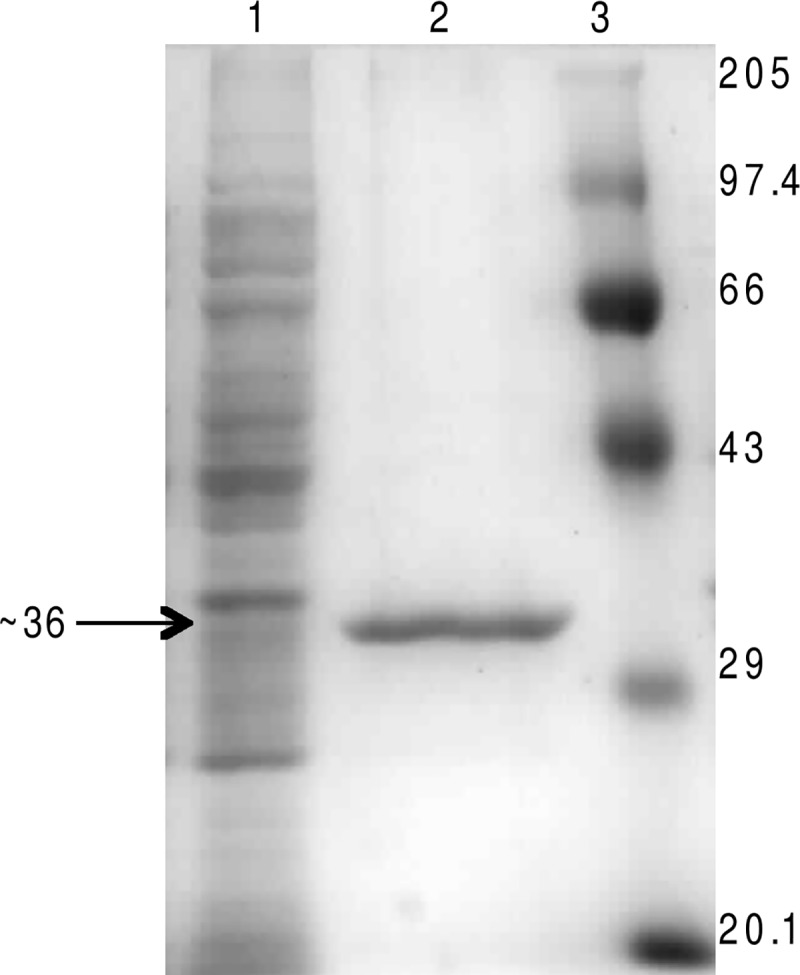
Purification of H3634 by Ni-NTA affinity chromatography. Cells were autoinduced to express H3634 as described in Materials and Methods. Cell lysates were loaded onto Ni-NTA columns and eluted with imidazole buffer. Cell lysate (lane 1), purified H3634 (lane 2) and molecular weight markers (lane 3). Molecular weights are in kDa. 10 μg of protein was loaded in each lane

The recombinant protein H3634 was expressed in *E*. *coli* BL21(DE3)pLysS growing in Auto-Induction medium at 25^°^C for 12 h under aerobic conditions. The level of expression of the recombinant protein is low under these conditions and the band cannot be visualised on a SDS-PAGE gel. Ni-NTA affinity chromatography of the soluble fractions obtained from cell lysates under these conditions led to the purification of H3634 (Fig 2). The yield of the Ni-NTA affinity purified protein was ~500 μg per litre of culture.

Expression of H3634 was also attempted in other strains of *E*. *coli* such as BL21(DE3), BL21-CodonPlus(DE3)-RIPL, OverExpressC41(DE3), OverExpressC43(DE3)pLysS at different temperatures (37, 25, 20, 16^°^C) and for varying durations (3–6 h) in AIM-LB or in LB broth in presence of varying concentrations of IPTG (0.1–1 mM). Protein expression was also attempted in *M*. *smegmatis* mc^2^ 4517 grown in 7H9/ADC/TW broth in presence of IPTG and acetamide. However, none of these approaches led to expression of H3634 in the soluble form.

### Epimerase activity of H3634

**Fig 3:**
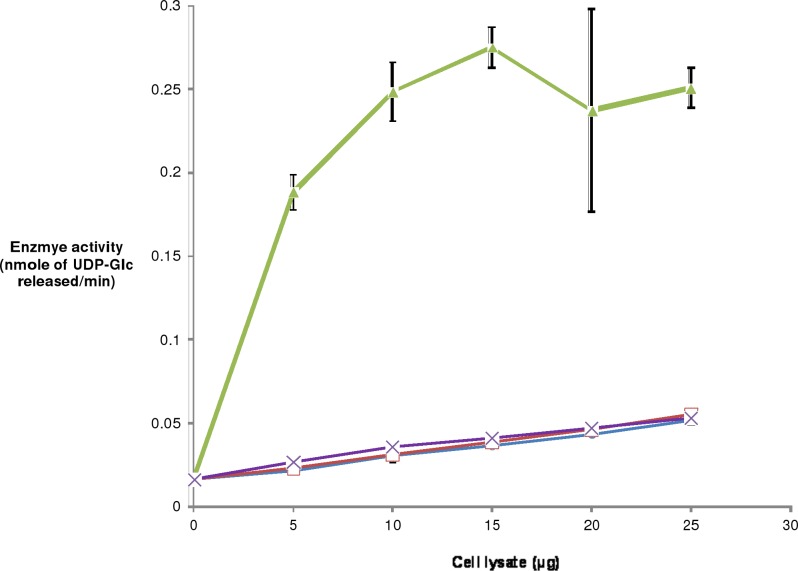
UDP-Gal 4-epimerase activity in lysates of cells containing pY3634 or negative controls. Reaction mixture containing 10mM Tris-HCl, pH 8.0, 1mM UDP-Gal, 0–30μg total protein was incubated at 37°C for 2h. Glucose was released from the nucleotide-sugar and assayed by the GOD-POD coupled reaction [21]. Epimerase activity of lysates containing no plasmid (blue circles), parent vector pYUB28b (red squares), pY3634 (green triangles) or pY3634Rev (violet cross).

**Fig 4:**
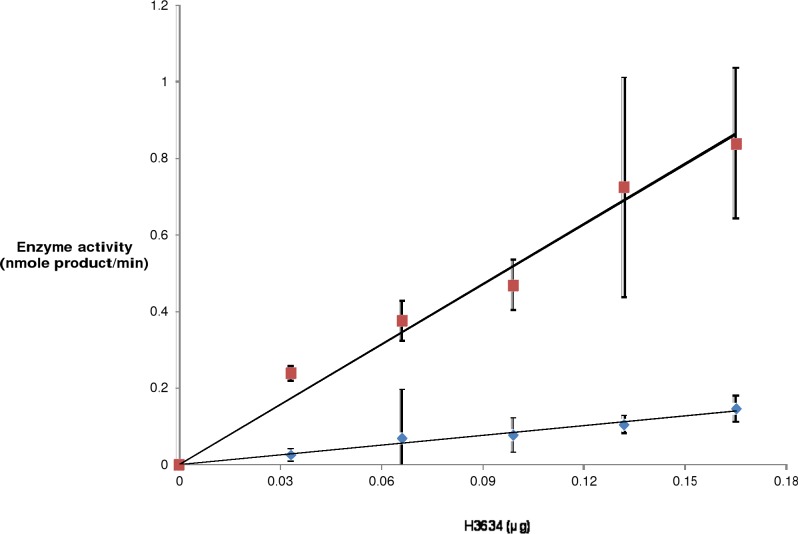
UDP-Gal/Glc epimerase activity of purified H3634 by GOD-POD assay. Reaction mixture containing 10mM Tris-HCl, pH 8.0, 2mM UDP-Glc/Gal, 0–0.165μg total protein was incubated at 37°C for 2h. Glucose was released from the nucleotide-sugar and assayed by the GOD-POD coupled reaction [21]. Epimerase activity of H3634 with UDP-Gal (red squares) or UDP-Glc as substrate (blue diamonds).

The GOD-POD assay provides a rapid and easy method to determine epimerase activity. This assay method was used to demonstrate that the protein expressed from the clone pY3634 has epimerase activity. Cell lysates were assayed for epimerase activity using 1 mM UDP-Gal as the substrate. Epimerase activity increased with increasing amounts of cell lysate of pY3634 whereas no epimerase activity was observed from the cell lysate of pY3634Rev or those containing parent vector pYUB28b or no plasmid (Fig 3). This suggested that epimerase activity is derived from the protein H3634 and not from the *E*. *coli* host. To confirm this observation, H3634 was purified from the cell lysates. Purified H3634 was tested for epimerase activity using the GOD-POD assay. It was observed that the epimerization reaction is catalyzed with both UDP-Gal and UDP-Glc as substrates. Varying amounts of protein were added to the reaction mixture containing UDP-Gal or UDP-Glc, and in both the cases activity increased with increasing amounts of H3634 (Fig 4). Thus, the protein H3634 is observed to have epimerase activity when UDP-Gal or UDP-Glc is used as substrate. Specific activity of purified H3634 with UDP-Gal was found to be 7.2 μmoles/min/mg and with UDP-Glc, it is 0.78 μmoles/min/mg. Thus there is a 10-fold difference in the specific activity of H3634 for UDP-Gal from that for UDP-Glc.

### Reverse phase HPLC for assaying UDP-Gal/Glc epimerase activities of H3634

**Fig 5:**
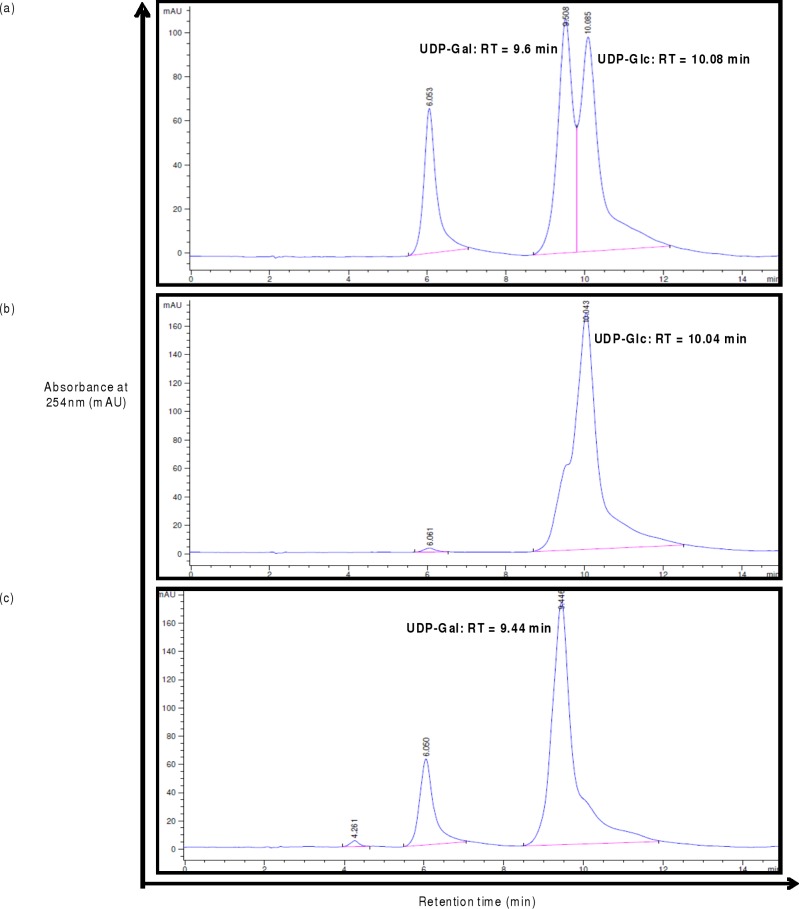
HPLC profiles for the UDP-Gal/Glc epimerase activity of H3634. UDP-Gal to UDP-Glc activity with 120ng H3634 (a), UDP-Glc to UDP-Gal activity with 300ng H3634 (b), UDP-Gal to UDP-Glc activity with 300ng of heat inactivated H3634 (c). All reaction mixtures were incubated at 37^°^C for 1h.

**Fig 6:**
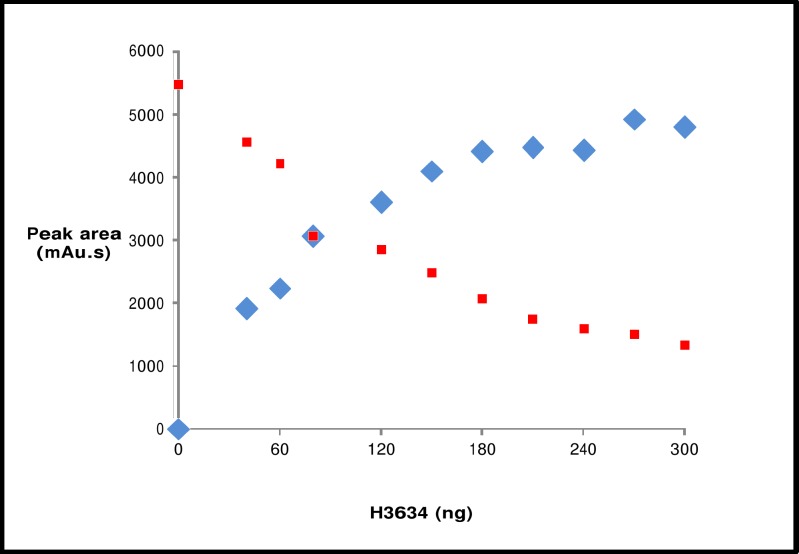
HPLC profile of UDP-Gal epimerase activity with increasing amounts of H3634. Area under UDP-Gal (red squares) and UDP-Glc (blue diamonds) peaks plotted against the amount of protein.

Reverse phase HPLC was used to further confirm UDP-Gal/Glc epimerization activity of H3634. Comparison of the elution profile of enzyme reaction mixture with the standard UDP-Gal and UDP-Glc profiles confirmed the identity of the reaction products (Fig 5). Elution profiles of reaction mixtures containing different amounts of H3634 incubated with UDP-Gal, indicated that UDP-Glc formation increased with increasing amounts of H3634, with a concomitant decrease of UDP-Gal (Fig 6). This established that H3634 UDP-Glc/Gal epimerase activity, as also seen in the GOD-POD assay.

### Analysis by HPLC of epimerase activity of H3634 using N-acetylated substrates

**Fig 7:**
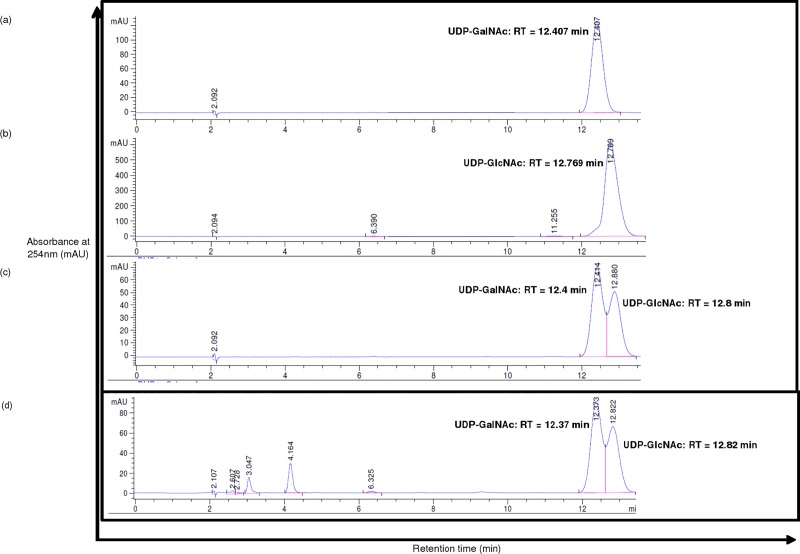
HPLC profiles for 1mM UDP-GalNAc (a), 1mM UDP-GlcNAc (b), 100μM mixture of UDP-GalNAc and UDP-GlcNAC (c), and reaction mixture containing 500μM UDP-GalNAc and 500ng of H3634 incubated for 7.5h at 37°C (d).

**Fig 8:**
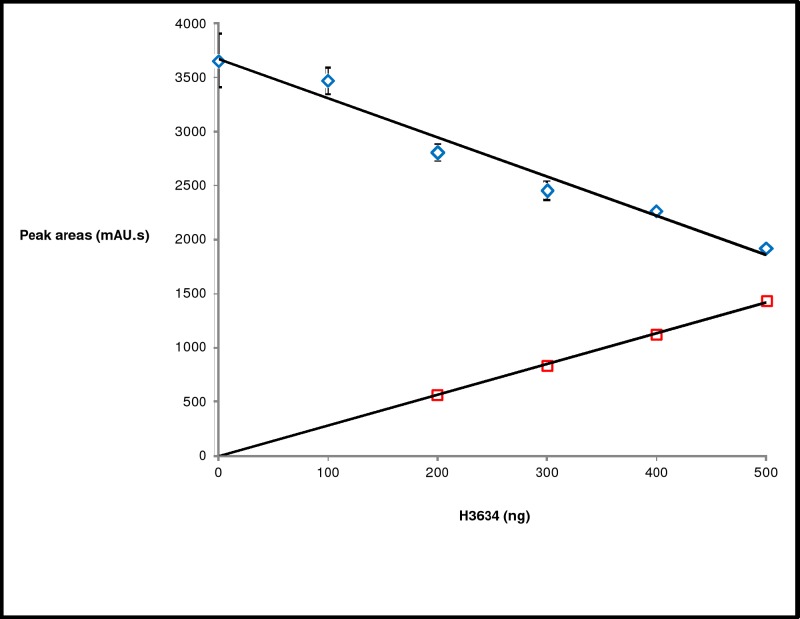
HPLC profiles of UDP-GalNAc 4-epimerase activity with increasing amounts of H3634. Area under UDP-GalNAc (blue diamonds) and UDP-GlcNAc (red squares) peaks plotted against the amount of protein.

*C*. *jejuni* Gne, homolog of Rv3634c, has epimerase activity with both UDP-Gal and UDP-GalNAc. To examine if H3634 has epimerase activity on UDP-GalNAc as well, various amounts of H3634 were incubated with UDP-GalNAc and product formation was monitored by HPLC. Retention times of the substrate and product were compared with those of commercially available UDP-GalNAc and UDP-GlcNAc (Fig 7). It was observed that the amount of UDP-GlcNAc formed increases with increasing amounts of the protein H3634 (Fig 8). This confirmed that H3634 has UDP-GalNAc epimerase activity. To assay for the activity of H3634 with UDP-GlcNAc as the substrate, the reaction mixture was incubated for 12 h at 37^°^C and HPLC was used to detect the product. A small shoulder was observed with the substrate peak. The retention time of the shoulder coincides with the product peak but it could not be quantified (data not shown). The ability of H3634 to utilize UDP-GlcNAc as substrate could not be demonstrated categorically.

### Mutation analysis of Rv3634c

**Fig 9:**
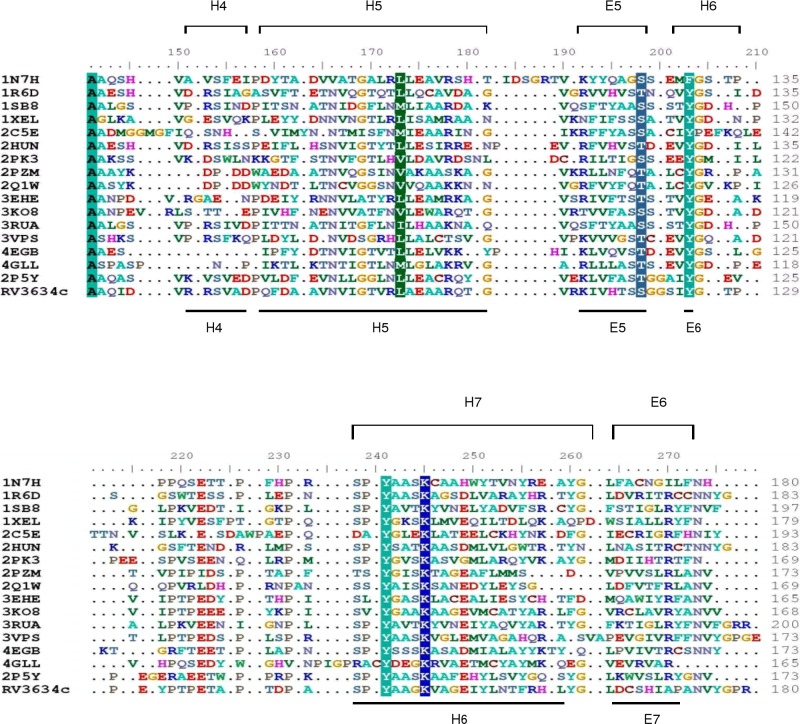
Multiple sequence alignment of NAD binding domains of nucleotide sugar epimerases. Sequences were aligned based on 3-D structure superposition using UDP-Glc 4-epimerase (2P5Y) from *T*. *thermophilus* as reference. Only a part of the alignment is shown. Conserved residues are shaded and dots indicate gaps. Secondary structural elements, based on crystal structures of the homologs, are shown on top of each alignment block. Predicted secondary structural elements of Rv3634c are shown at the bottom of each alignment block. Organism and protein names corresponding to the PDB entries used in the alignment are given in S2 Table. Source organism of each protein is mentioned as follows: 1N7H –*Arabidopsis thaliana*, 1R6D - *Streptomyces venezuelae*, 1SB8—*Pseudomonas aeruginosa*, 1XEL*—Escherichia coli*, 2C5E *- Arabidopsis thaliana*, 2HUN*—Pyrococcus horikoshii OT*, 2PK3—*Aneurinibacillus thermoaerophilus*, 2PZM*—Bordetella bronchiseptica*, 2Q1W *- Bordetella bronchiseptica*, 3EHE—*Archaeoglobus fulgidus*, 3KO8*—Pyrobaculum calidifontis*, 3RUA*—Plesiomonas shigelloides*, 3VPS*—Streptomyces chartreusis* NRRL 3882, 4EGB*—Bacillus anthracis* str. Ames, 4GLL*—Homo sapiens*, 2P5Y - *Thermus thermophilus* HB8

Nucleotide sugar epimerases use serine/threonine, tyrosine and lysine as catalytic residues (Fig 9) [23][24][25]. The first step in the reaction mechanism is abstraction of a hydride. This is followed by the rotation of sugar moiety and transfer of proton back to the sugar giving rise to the epimerised product [8]. Based on a multiple sequence alignment of epimerases with known 3-D structures, residues Ser121, Tyr146 and Lys150 in Rv3634c were identified as putative catalytic residues (Fig 9). In order to characterize essentiality of these residues for the epimerase activity of Rv3634c, Ser121, Tyr146 and Lys150 were mutated to alanine, phenylalanine and arginine, respectively.

**Fig 10:**
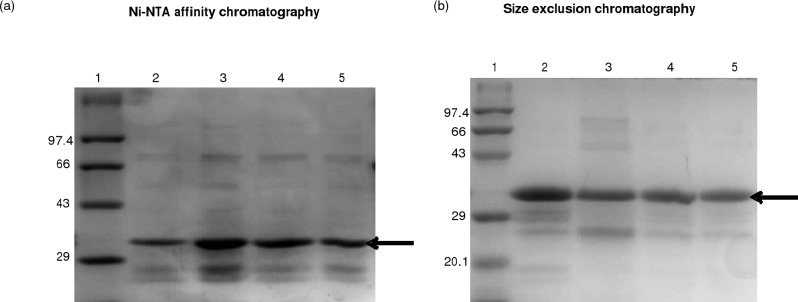
Purification of WT and mutant proteins by Ni-NTA affinity chromatography (a) and size exclusion chromatography (b). Molecular weight markers (Lane 1), elution fractions of WT H3634 (Lane 2), S121A (Lane 3), Y146F (Lane 4) and K150R (Lane 5).

In the case of Ser and Tyr, the suggested role involves the hydroxyl (-OH) group [26]. Hence, these residues were mutated in such a way so as to remove the hydroxyl group. If these are indeed the catalytic residues, such a mutation is expected to affect catalysis without destabilizing the folded state of the protein. The suggested role of lysine in *E*. *coli* UDP-Glc/Gal epimerase is to provide positive electrostatic potential so as to decrease the pKa of Tyr [25]. One possibility was to substitute it with negatively charged residue Asp or Glu. However, if the resultant mutant is inactive, it will not be possible to unambiguously infer whether Lys has a catalytic or a structural role. In addition, Lys has four -CH_2_ groups that can contribute to the hydrophobic packing of the protein. In light of this, Lys was mutated to Arg with the anticipation that the mutation will have either no effect on activity or partial effect on activity, as it provides a positive charge. The S121A, Y146F and K150R mutants of H3634 were generated by PCR based site directed mutagenesis using the primers PSP025/06, PSP027/028 and PSP029/030, respectively, as described in Materials and Methods. Mutations were confirmed by DNA sequencing. Mutant proteins were expressed under identical conditions as that for the wild type. The solubility and Ni-NTA affinity purification profile (Fig 10A) of the mutants were found to be same as those for WT. Ni-NTA purified proteins were further subjected to size exclusion chromatography (Fig 10B) and then used for further biochemical and biophysical characterization. Size exclusion chromatography yielded the WT and mutant proteins in pure form. Yield of the purified proteins after size exclusion chromatography was ~150–250 μg per litre of culture. The low molecular weight bands found on SDS-PAGE are probably due to degradation of the purified protein (Fig 10B).

### Epimerase activity of H3634 mutants by GOD-POD assay

**Fig 11:**
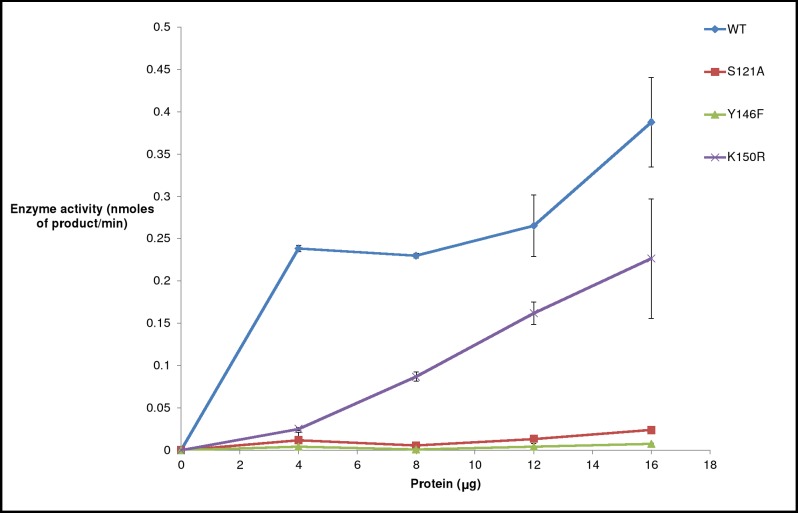
UDP-Gal 4-epimerase activity of purified WT and mutant H3634 proteins. Activities determined by GOD-POD assay [21].

To determine the effect of mutation on the epimerase activity of H3634, purified WT and mutant H3634 proteins were assayed with UDP-Gal as the substrate by GOD-POD assay. This revealed that S121A and Y146F mutants were completely inactive whereas K150R showed partial loss of activity (Fig 11). The highest specific activity of the size exclusion purified K150R mutant was found to be 0.3 μmoles/min/mg, whereas that of the WT was 2.35 μmoles/min/mg. Thus, the mutation K150R led to about 85–90% loss of epimerase activity. The results suggested that S121 and Y146 are indispensable for epimerase activity of H3634, whereas in the case of K150R mutant, arginine may be able to partially substitute for lysine leading to a partially active enzyme. These results are in support of the existing reports for homologous proteins, in which Ser and Tyr residues have been observed to be essential for epimerase activity [25][8]. These experiments also support the observation that the source of epimerase activity is indeed the recombinant protein H3634.

### Secondary structure analysis by CD spectroscopy

**Fig 12:**
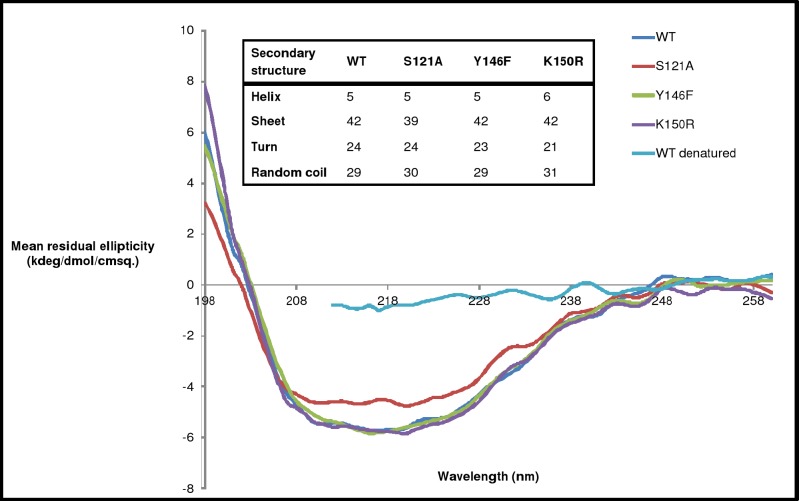
CD spectra for WT and mutant H3634 purified proteins. Percentages of helix and sheet content calculated by deconvulation of spectra are shown as inset.

To examine the effect of mutation on gross secondary structure content of H3634, CD spectroscopy was used. CD spectra of the mutant proteins were similar to that of the WT indicating that there are no gross changes in the secondary structures of the mutants (Fig 12). Deconvolution of spectra showed that the secondary structure content of the mutant proteins is comparable to that of the WT. This suggested that the mutated residues play an important role in catalysis or binding, as the activity is lost without any significant change in secondary structure.

### Addition of NAD^+^ partially restores epimerase activity of K150R

**Fig 13:**
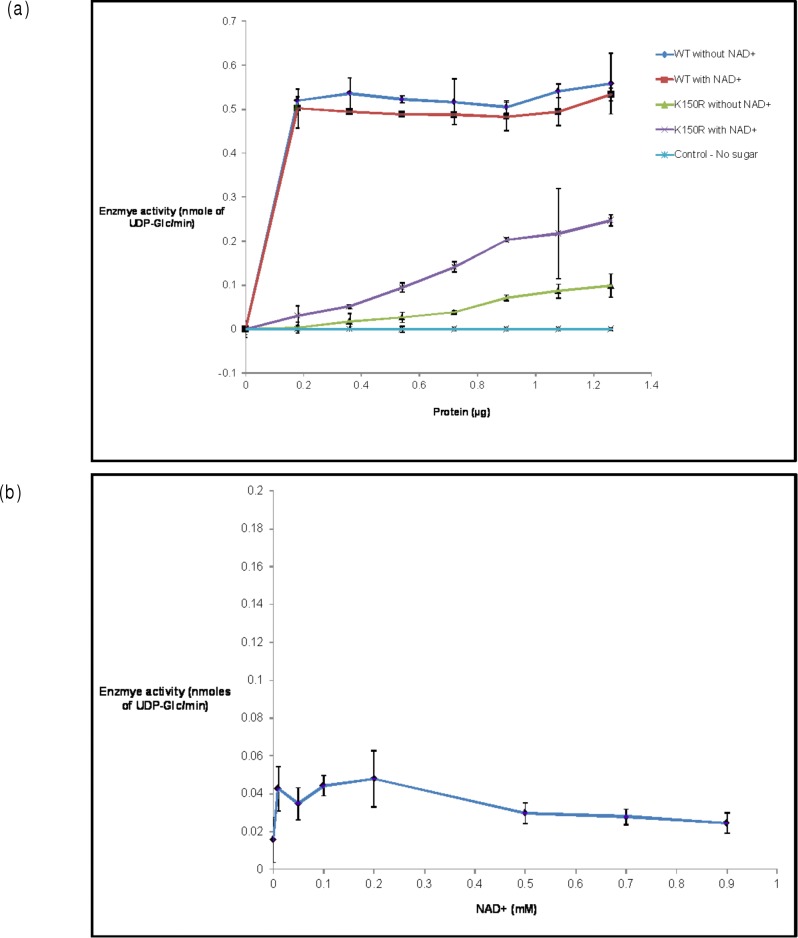
(a) UDP-Gal 4-epimerase activity of WT and K150R purified proteins in presence or absence of 100μM NAD^+^. (b) UDP-Gal 4-epimerase activity of K150R with varying concentrations of NAD^+^. Reaction mixture contained 360ng K150R, 1mM UDP-Gal and NAD^+^ at varying concentrations.

Lys has been reported to be a part of catalytic site in many of the epimerases [8]. It may also be involved in NAD+ binding. In order to examine the possible role of K150 on NAD^*+*^ binding, epimerase activity of the mutant was determined with and without NAD^*+*^ added to the reaction mixture. The K150R mutant showed higher activity with UDP-Gal as the substrate in the presence of added NAD^*+*^, as compared to in its absence (Fig 13A). Addition of NAD^+^ could increase the epimerase activity of K150R mutant by ~2.5 folds. This showed that K150 may be involved in NAD^*+*^ binding (Fig 13B). Similar experiments with other two mutants did not show activity even in the presence of NAD^+^ (data not shown).

### Oligomerization status of proteins by size exclusion chromatography

**Fig 14:**
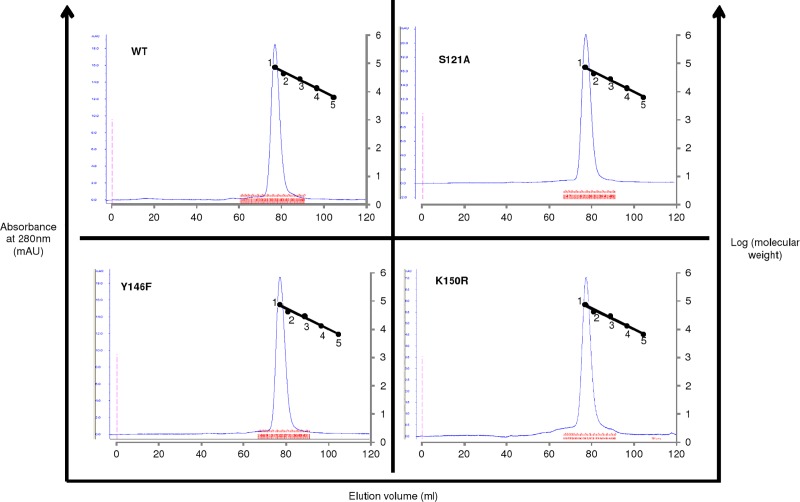
Elution profiles of WT and mutant H3634 on gel filtration column. Calibration curve was plotted using conalbumin [75kDa] (1), ovalbumin [44kDa] (2), carbonic anhydrase [29kDa] (3), ribonuclease A [13.7kDa] (4) and aprotinin [6.5kDa] (5).

Most of the reported epimerases exist as dimers in solution [8][27]. To examine the oligomeric status of H3634 and the effect of mutation on the quaternary structure of H3634, WT and mutant H3634 proteins were subjected to size exclusion chromatography. All four proteins eluted at the same position i.e. ~78 min. Comparison of the elution profiles with the calibration curve revealed that all four proteins existed as dimers of size ~67 kDa (Fig 14). This showed that none of the mutations affected the quaternary structure of the protein. Hence, loss of activity is not due to loss in the quaternary structure of the recombinant protein.

## Discussion

In organisms belonging to the genus *Mycobacterium*, UDP-Glc/Gal epimerase activity has been reported only from *M*. *smegmatis* so far [13]. In the comparative genomics study that used the N-glycosylation pathway enzymes of *C*. *jejuni* as reference sequences [28][29], only Rv3634c was identified as the homolog of Gne (i.e., UDP-Glc/Gal and UDP-GalNAc/GlcNAc 4-epimerase). This study has focussed on the experimental demonstration of the epimerase activity of recombinant Rv3634c using UDP-Gal/Glc and UDP-GalNAc as substrates *in vitro*. The *Rv3634c* gene was cloned, expressed as a His-tagged protein in *E*. *coli* and the recombinant protein was purified using Ni-NTA affinity chromatography. Recombinant Rv3634c has been shown to have UDP-Gal/Glc 4-epimerase activity by GOD-POD assay and HPLC. Further, it has been established by HPLC that recombinant Rv3634c possesses UDP-GalNAc 4-epimerase activity, *in vitro*.

Residues essential for the structure and/or function of a protein tend to be conserved. Mutation of such residues leads to loss of function with or without concomitant loss of three-dimensional structure. Change or loss of activity on mutation without change in the secondary, tertiary and/or quaternary structure of the protein, indicates a direct role of the mutated residue in catalysis and/or binding. However, if there is any change in the structure of protein on mutation, it reveals the importance of the mutated residue in maintaining the structure of the protein and hence, an indirect role in the function of the protein. Thus, a comparison of overall structures of the mutants and the WT proteins is important to understand the structure-function relationship in recombinant Rv3634c. It was observed that the mutating Ser121 to Ala or Tyr146 to Phe leads to complete loss of epimerase activity of recombinant Rv3634c whereas mutating Lys150 to Arg leads to partial loss but none of these mutations affect the secondary or quaternary structure of the protein. Thus, the loss in epimerase activity of recombinant Rv3634c may be due to loss of functional groups essential for catalysis and/or binding of substrate/NAD^+^. Based on the data for other epimerases in the literature, probable roles can be assigned to the mutated residues. Thus, it can be inferred that in S121A and Y146F mutants, the loss of hydroxyl groups essential for proton abstraction from the nucleotide sugar, leads to loss of function. The partial activity seen in K150R mutant is probably due to the substitution of functional group of Lys by Arg, as both have similar charge on them.

Recombinant Rv3634c does not require added NAD^+^ for UDP-Gal/Glc or UDP-GalNAc epimerase activity, *in vitro*. Addition of NAD^+^ to the reaction mixture with the WT protein did not increase the epimerase activity, indicating that NAD^+^ is bound to the WT protein and hence, is co-purified with the protein. Since mutations are near the probable NAD^+^ binding domain, it was anticipated that these mutations may lead to some subtle local changes, which may not be detected by CD spectra. If the mutations affect binding of the cofactor NAD^+^, it may be lost from the protein during purification. To examine this possibility, the partially active mutant K150R was tested for increase in residual activity with the addition of NAD^+^. Indeed, addition of NAD^+^ increased the epimerase activity of K150R protein confirming that the K150 residue is essential for NAD^+^ binding. If binding alone is affected by the mutation, addition of higher amounts of NAD^+^ should restore the activity of K150R to the WT level. However, the addition of increasing amounts of NAD^+^ could not rescue the complete activity of K150R, thus suggesting that K150 is essential for not just binding but also for the catalysis in the epimerase enzyme. This finding corroborates the existing data for the reported epimerases. The S121A and Y146F mutants were completely inactive, irrespective of the presence or absence of exogenous NAD^+^. Nevertheless, loss of activity without loss of structure in these mutants also suggests that they are essential for binding and/or catalysis, as in the case of reported UDP-Gal/Glc epimerases from various organisms [30][31].

### Cellular location of Rv3634c from high throughput studies

To understand the function of *Rv3634c* in the organism, it is important to define its role at cellular level in addition to its molecular function. This has been achieved to some extent by various proteomics, transcriptomics studies. The proteome of *M*. *tuberculosis* H37Rv culture grown on modified Sauton medium analysed by 2-D PAGE and MS/MS revealed that Rv3634c protein is present in cell lysates but not in the spent medium, indicating that this protein is not secreted [5]. Expression of Rv3634c was found in the membrane fraction of MtbH37Rv grown in defined medium, indicating that the Rv3634c protein may be membrane associated [4]. Rv3634c was found in whole cell lysates as well as in lipid phases of the triton X-114 extract but not in the culture filtrates. Also, Rv3634c does not have any transmembrane helices and its hydropathy score is low. This indicates that Rv3634c is a soluble protein, but may be co-localised with some membrane proteins as part of its function [32][33]. Proteomics and transposon mutagenesis studies indicate that Rv3634c is neither a membrane protein [4][34] nor a part of the cell wall proteome [35].

### Possible biological role of *Rv3634c*

As discussed above, *Rv3634c* encodes a soluble protein whose molecular activity is UDP-Glc/Gal and UDP-GalNAc epimerase. It is essential to determine the biological role of this protein at pathway level. In *Drosophila melanogaster*, *Campylobacter jejuni* NCTC 11168, *Lactococcus lactis* and *Candida albicans*, UDP-Gal 4-epimerase has been shown to have both catabolic and anabolic roles. The catabolic role is in the utilization of galactose as a carbon/energy source [36]. In *D*. *melanogaster*, knockdown of the *galE* gene was found to be embryonic lethal as well as specific to the stage of growth in a sex dependent manner. In *C*. *jejuni*, disruption of the gene results in structural alterations of lipooligosaccharide as well as the capsule [29][37]. In *L*. *lactis*, disruption of *galE* results in impaired extracellular polysaccharide production [38]. In *C*. *albicans*, disruption of the *gal10* gene impairs cell wall integrity and resistance to stress [39]. Thus, UDP-Gal/Glc epimerase has an important role in all the organisms studied and is indispensable for their growth and/or metabolism.

In contrast to the above mentioned organisms, *E*. *coli* O86:B7 has three UDP-sugar 4-epimerase genes, out of which *galE* is a part of galactose metabolism operon and *gne1* and *gne2* belong to the O-antigen gene cluster. Both Gne1 and Gne2 catalyze the interconversion of UDP-Gal/Glc and UDP-GalNAc/GlcNAc, although their activities are not comparable to each other. It has been demonstrated that both *gne1* and *gne2* are required for UDP-GalNAc biosynthesis but not for UDP-Gal biosynthesis. Strains lacking *gne1* and *gne2* are found to have abnormal LPS [40].

In the *M*. *tuberculosis* whole genome sequencing project, four ORFs were found to be homologous to *rmlB*. These are *Rv3464*, *Rv3634c*, *Rv3784* and *Rv3468c*. *In vitro* assays have shown that, Rv3464 has dTDP-Glc dehydratase (RmlB) activity whereas Rv3784 and Rv3468c do not have this enzyme activity [12]. The molecular functions of Rv3784 and Rv3468c have not been experimentally determined so far and hence, it is not clear whether Rv3634c is the only UDP-Gal/GalNAc 4-epimerase or there are other gene products with this enzyme activity. Functional network analysis by global subcellular protein profiling of *M*. *tuberculosis* H37Rv grown for 14 d on glycerol alanine salts medium showed that Rv3634c is not one of the 1044 proteins identified by tandem mass spectroscopy [41]. *Rv3634c* is not expressed even during the log phase of *M*. *tuberculosis* H37Rv grown on 7H9 medium as revealed by gene expression data based on Affymetrix gene chips [42]. *Rv3634c* is not expressed even in chemostat-cultures of *M*. *tuberculosis* H37Rv grown on 7H9 medium [43]. In contrast, a transposon site hybridization study identified *Rv3634c* as one of the 614 genes required for optimal growth on 7H9 medium [34]. High-density mutagenesis and deep sequencing studies predicted that *Rv3634c* is not an essential gene for growth on cholesterol, or for growth in mice models of infection [35][44].

In a transcriptomic study of nutrient starvation model of *M*. *tuberculosis* H37Rv, *Rv3634c* RNA expression was found to be about four fold upregulated after 4 h of starvation as compared to the zero time point. But this upregulation was not seen after 24 h or 96 h [2]. Rv3634c expression was found to be upregulated by 2.25 folds in *M*. *tuberculosis* H37Rv cells infecting the macrophages as compared to that in *in vitro* culture. This upregulation was absent in the *sigE* mutant infecting the macrophages [45]. This indicates that *Rv3634c* is in some way involved in intracellular survival of *M*. *tuberculosis* and is regulated by the sigma factor *sigE*. This sigma factor controls the extracytoplasmic function genes. Most of the genes regulated by this sigma factor are involved in cell wall and membrane component synthesis and maintenance [46]. Thus, it is possible that even *Rv3634c* is involved in the biosynthesis of glycoconjugates required for cell wall synthesis. Also, it has been reported that *Rv3631* and *Rv3632* are involved in the synthesis of galactosamine substituent of arabinogalactan in Mtb [47]. The close proximity of these genes to *Rv3634c* suggests that *Rv3634c* could be involved in maintaining the UDP-Gal/Glc and UDP-GalNAc/GlcNAc pool for the biosynthesis of these cell wall glycoconjugates. The present study was aimed at the experimental determination of the molecular function of *Rv3634c*. As mentioned in the Introduction, complete description of the function of a protein requires specifying its biological role as well. One of the biological functions ascribed to UDP-Gal 4-epimerase is in the catabolism of galactose through the Leloir pathway [36]. In addition, UDP-Gal 4-epimerase has been ascribed an anabolic role in providing Gal and GalNAc for the biosynthesis of extracellular glycans. Given the structural differences in such glycans across strains, species and genera, extrapolating the specific anabolic role can at best be tentative.

## Supporting information

S1 Table Oligonucleotide primers used for amplification and mutagenesis of Rv3634c. (PDF)

S2 Table Homologs of Rv3634c whose 3D structures are known. (PDF)

S1 Fig Reaction scheme for epimerase and GOD-POD coupled assay. (PDF)

S2 Fig Strategy used for cloning the gene Rv3634c in the plasmid pYUB28b. The gene was inserted between the NdeI and BamHI sites to express protein with N-terminal His_6_ tag.
(PDF)

## Abbreviations

AIM-LB: Auto induction medium-LB broth with trace elements
BLAST: Basic local alignment search algorithm
dTDP: Deoxythymidine diphosphate
Gal: D-Galactopyranose
GDP: Guanosine diphosphate
Glc: D-Glucopyranose
GlcA: D-Glucuronic acid
GlcNAc: 2-Deoxy-2-acetamido-D-glucopyranose
GOD-POD: Glucose oxidase–peroxidase
Man: D-Mannose
MSA: Multiple sequence alignment
Mtb: *Mycobacterium tuberculosis* H37Rv
NAD: Nicotinamide adenine dinucleotide
Ni-NTA: Nickel-nitrilotriacetic acid
PDB: Protein databank
PMSF: Phenyl methyl sulfonyl fluoride
SDR: Short-chain dehydrogenase / reductase
UDP: Uridine diphosphate
X-Gal: 5-Bromo-4-chloro-3-indolyl-β-D-galactopyranoside
