# Supplementary material for: Rv3634c from Mycobacterium tuberculosis H37Rv encodes an enzyme with UDP-Gal/Glc and UDP-GalNAc 4-epimerase activities
Source: PLoS One. 2017 Apr 12;12(4):e0175193. doi: 10.1371/journal.pone.0175193 (PMC5389812; doi:10.1371/journal.pone.0175193)
Supplement: S1 Table — (PDF) [file pone.0175193.s001.pdf]

S1 Table.

Primer Code	Primer sequence (5′-3′)	Properties
PSP021	ATGC **AAG CTT** AGAC **CAT ATG** CGC GCA CTG GTC ACT GG	-HindIII-SPACER-NdeI-MRALVT-
PSP024	ATGC **GGA TCC** TCA ATC AGT GTG CTT GTG CCG GAA GTA	-YFRHKHTD-STOP-BamHI-
PSP025	GTG CAC ACC TCC GCG GGA GGA TCG ATC	Forward primer for S121A
PSP026	GAT CGA TCC TCC CGC GGA GGT GTG CAC	Reverse primer for S121A
PSP027	GAT CCG GCC TCG CCG TTT GCC GCG GGC	Forward primer for Y146F
PSP028	GCC CGC GGC AAA CGG CGA GGC CGG ATC	Reverse primer for Y146F
PSP029	CC GCG GGC AGA GTG GCC GGC GAA ATC	Forward primer for K150R
PSP030	GTA GAT TTC GCC GGC CAC TCT GCC CGC GGC	Reverse primer for K150R
