# Supplementary material for: Rv3634c from Mycobacterium tuberculosis H37Rv encodes an enzyme with UDP-Gal/Glc and UDP-GalNAc 4-epimerase activities
Source: PLoS One. 2017 Apr 12;12(4):e0175193. doi: 10.1371/journal.pone.0175193 (PMC5389812; doi:10.1371/journal.pone.0175193)
Supplement: S2 Table — (PDF) [file pone.0175193.s002.pdf]

S2 Table.

Organism	Protein	SwissProt/ PDB id	Length (AA)	Identity, Similarity (%)	Alignment coverage¶	E-value
**Experimentally characterized homologs**						
Aneurinibacillus thermoaerophilus	GDP-6-deoxy-D-*lyxo*-4-hexulose reductase	Q6T1X6 2PK3	309	26, 43	98, 99	7.0 E-27
Arabidopsis thaliana	GDP-Man 3,5-epimerase	Q93VR3 2C5E	377	25, 43	99, 83	9.0 E-20
Arabidopsis thaliana	GDP-Man 4,6-dehydratase	P93031 1N7H	372	29, 44	97, 86	2.0 E-24
Escherichia coli	UDP-Gal 4- epimerase	P09147 1XEL	338	33, 45	92, 93	2.0 E-33
Homo sapiens	UDP-GlcA decarboxylase	Q8NBZ7 4GLL	420	29, 49	98, 72	5.0 E-42
Plesiomonas shigelloides	UDP-GalNAc 4-epimerase	Q7BJX9 3RUA	345	32, 49	97, 92	4.0 E-44
Pseudomonas aeruginosa	UDP-GlcNAc 4-epimerase	Q8KN66 1SB8	341	33, 49	97, 93	4.0 E-46
Pyrobaculum calidifontis	UDP-Gal 4- epimerase	A3MUJ4 3KO8	312	35, 48	97, 97	1.0 E-45
Streptomyces venezuelae	dTDP-Glc 4,6-dehydratase	Q9ZGH3 1R6D	337	35, 48	98, 93	2.0 E-48
Thermus thermophilus HB8	UDP-Glc 4-epimerase	Q5SKQ2 2P5Y	311	45, 57	98, 99	2.0 E-76
**Computationally annotated homologs**						
Archaeoglobus fulgidus	UDP-Glc 4-epimerase	O29886 3EHE	305	33, 50	96, 96	5.0 E-37
Bacillus anthracis str. Ames	dTDP-glucose 4,6-dehydratase	Q81TP0 4EGB	322	27, 47	98, 96	1.0 E-33
Bordetella bronchiseptica	Putative nucleotide sugar epimerase/dehydratase	O87987 2Q1W	313	34, 46	98, 94	2.0 E-33
Bordetella bronchiseptica	Putative nucleotide sugar epimerase/dehydratase	O87988 2PZM	310	28, 46	98, 95	3.0 E-31
Pyrococcus horikoshii OT	Hypothetical dTDP-glucose 4,6-dehydratase	O58151 2HUN	336	31, 49	98, 92	3.0 E-36
Streptomyces chartreusis NRRL 3882	NAD-dependent epimerase/dehydratase	E5KJ94 3VPS	321	29, 43	99, 93	5.0 E-28

¶ Percentage of the sequence (Rv3634c, homolog) that is covered by the alignment.
