# Supplementary figures and images for: Rv3634c from Mycobacterium tuberculosis H37Rv encodes an enzyme with UDP-Gal/Glc and UDP-GalNAc 4-epimerase activities
Source: PLoS One. 2017 Apr 12;12(4):e0175193. doi: 10.1371/journal.pone.0175193 (PMC5389812; doi:10.1371/journal.pone.0175193)

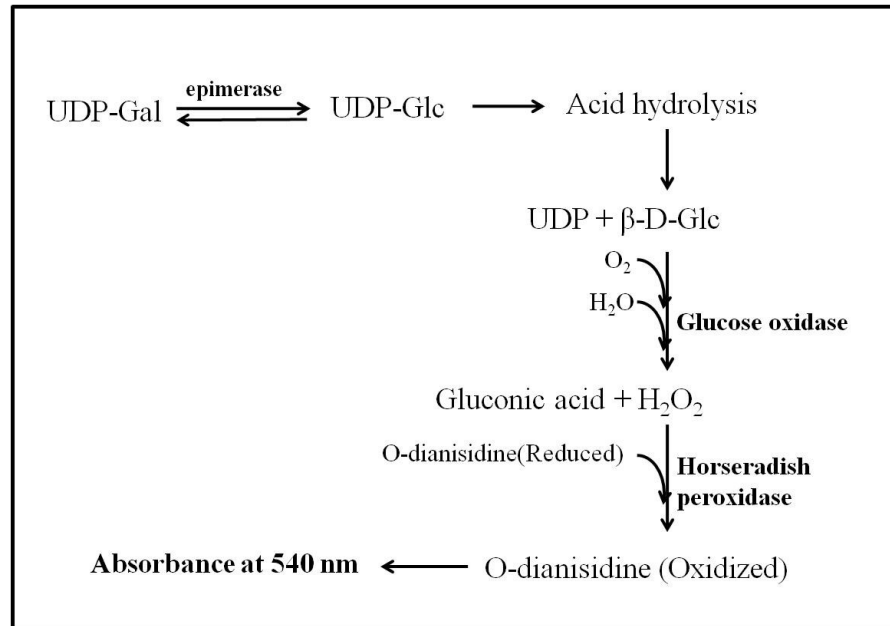

S1 Fig.

Supplement: S1 Fig — (PDF) [file pone.0175193.s003.pdf]

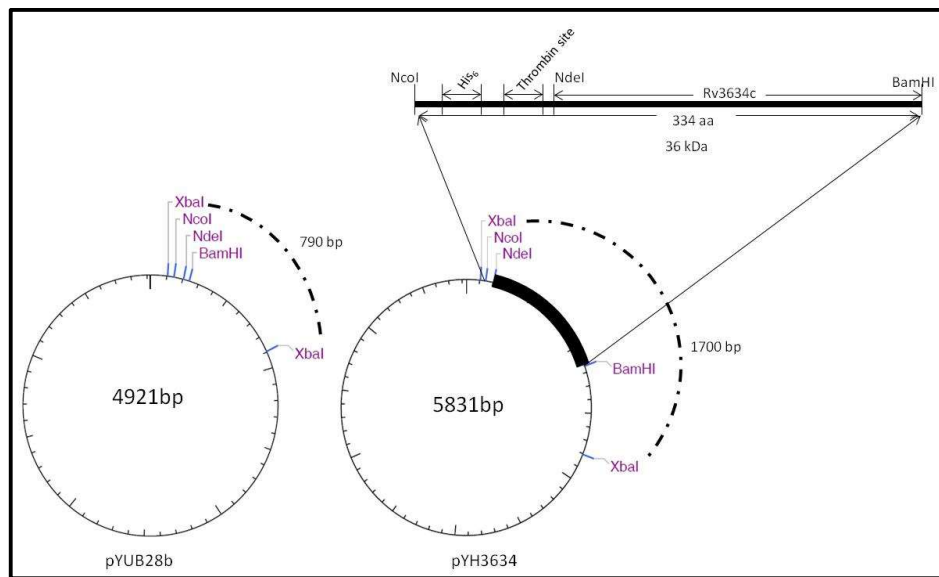

S2 Fig.

Supplement: S2 Fig — The gene was inserted between the NdeI and BamHI sites to express protein with N-terminal His6 tag. (PDF) [file pone.0175193.s004.pdf]
